# Causal effects of gut microbiota on chalazion: a two-sample Mendelian randomization study

**DOI:** 10.3389/fmed.2024.1411271

**Published:** 2024-06-04

**Authors:** Wenfei Zhang, Xingwang Gu, Qing Zhao, Chuting Wang, Xinyu Liu, Youxin Chen, Xinyu Zhao

**Affiliations:** ^1^Department of Ophthalmology, Peking Union Medical College Hospital, Chinese Academy of Medical Sciences & Peking Union Medical College, Beijing, China; ^2^Key Laboratory of Ocular Fundus Diseases, Chinese Academy of Medical Sciences, Beijing, China

**Keywords:** gut microbiota, chalazion, Mendelian randomization, inverse variance weighted, single nucleotide polymorphisms

## Abstract

**Purpose:**

To investigate the causal relationship between gut microbiota (GM) and chalazion through Mendelian randomization (MR) analysis.

**Methods:**

GM-related genome-wide association studies (GWAS) were obtained from the International Consortium MiBioGen. Genetic data for chalazion were sourced from the MRC Integrative Epidemiology Unit (IEU) Open GWAS database. Five MR methods, including inverse variance weighted (IVW), were employed to estimate causal relationships. Cochran’s Q test was used to detect heterogeneity, the MR-Egger intercept test and MR-PRESSO regression were utilized to detect horizontal pleiotropy, and the leave-one-out method was employed to validate data stability.

**Results:**

We identified 1,509 single nucleotide polymorphisms (SNPs) across 119 genera as instrumental variables (IVs) (*p* < 1 × 10^−5^). According to the inverse variance weighted (IVW) estimate, the Family XIII AD3011 group (OR = 1.0018, 95% CI 1.0002–1.0035, *p* = 0.030) and Catenibacterium (OR = 1.0013, 95% CI 1.0002–1.0025, *p* = 0.022) were potentially associated with increased risk of chalazion. Conversely, Veillonella (OR = 0.9986, 95% CI 0.9974–0.9999, *p* = 0.036) appeared to provide protection against chalazion. There was no evidence of heterogeneity or pleiotropy.

**Conclusion:**

This study uncovered the causal relationship between GM and chalazion, pinpointing Catenibacterium and Family XIII AD3011 group as potential risk contributors, while highlighting Veillonella as a protective factor. In-depth investigation into the potential mechanisms of specific bacteria in chalazion was essential for providing novel therapeutic and preventive strategies in the future.

## Introduction

Chalazion is a common lipogranulomatous inflammation of the eyelid originating from the meibomian or Zeis gland. The incidence of chalazion ranges from 0.2 to 6.04%, with a higher prevalence observed in children compared to adults (1, 2). The formation of chalazion is attributed to a variety of factors, including systemic disorders such as inflammatory bowel disease, depression, and anxiety; metabolic abnormalities like vitamin A deficiency, diabetes, and higher serum cholesterol levels; ocular factors such as meibomian gland disease (MGD), conjunctivitis, blepharitis, and dry eye; additional factors like race, ethnicity, as well as dietary factors (3, 4).

Human intestines harbor a vast array of microorganisms, which play an essential role in host health by adjusting the internal environment. Dysbiosis of the gut microbiota (GM) has been related to various ophthalmic diseases, including uveitis (5), corneal-conjunctival diseases like vernal keratoconjunctivitis and dry eye (6, 7), glaucoma, and retinal diseases (8–11). The interplay between GM and chalazion is intricate and influenced by numerous confounding factors, which has led to a scarcity of research elucidating their relationship. A previous prospective comparative pilot study indicated that supplementing with probiotics could lead to complete resolution of chalazion in a shorter period compared to medical treatment alone, suggesting a role of specific GM in chalazion. However, as noted in the article, the current data were limited by small sample sizes and follow-up duration, and the experimental conclusions were preliminary, precluding definitive conclusions (12, 13). Furthermore, considering the diversity of GM, it is challenging to determine which strains are truly beneficial for chalazion, posing a challenge to the selection of probiotics.

Utilizing summarized data from genome-wide association studies (GWAS) and prioritizing genetic variants closely linked to the exposure, Mendelian randomization (MR) furnishes robust evidence for causal effects of exposures on outcomes (14, 15). To date, there have been no MR studies exploring the association between GM and chalazion. We performed the MR analysis to demonstrate the causal impact of GM on chalazion, aiming to unveil novel avenues for preventive and therapeutic interventions.

## Method

### Assumptions and study design of MR

**Figure 1:**
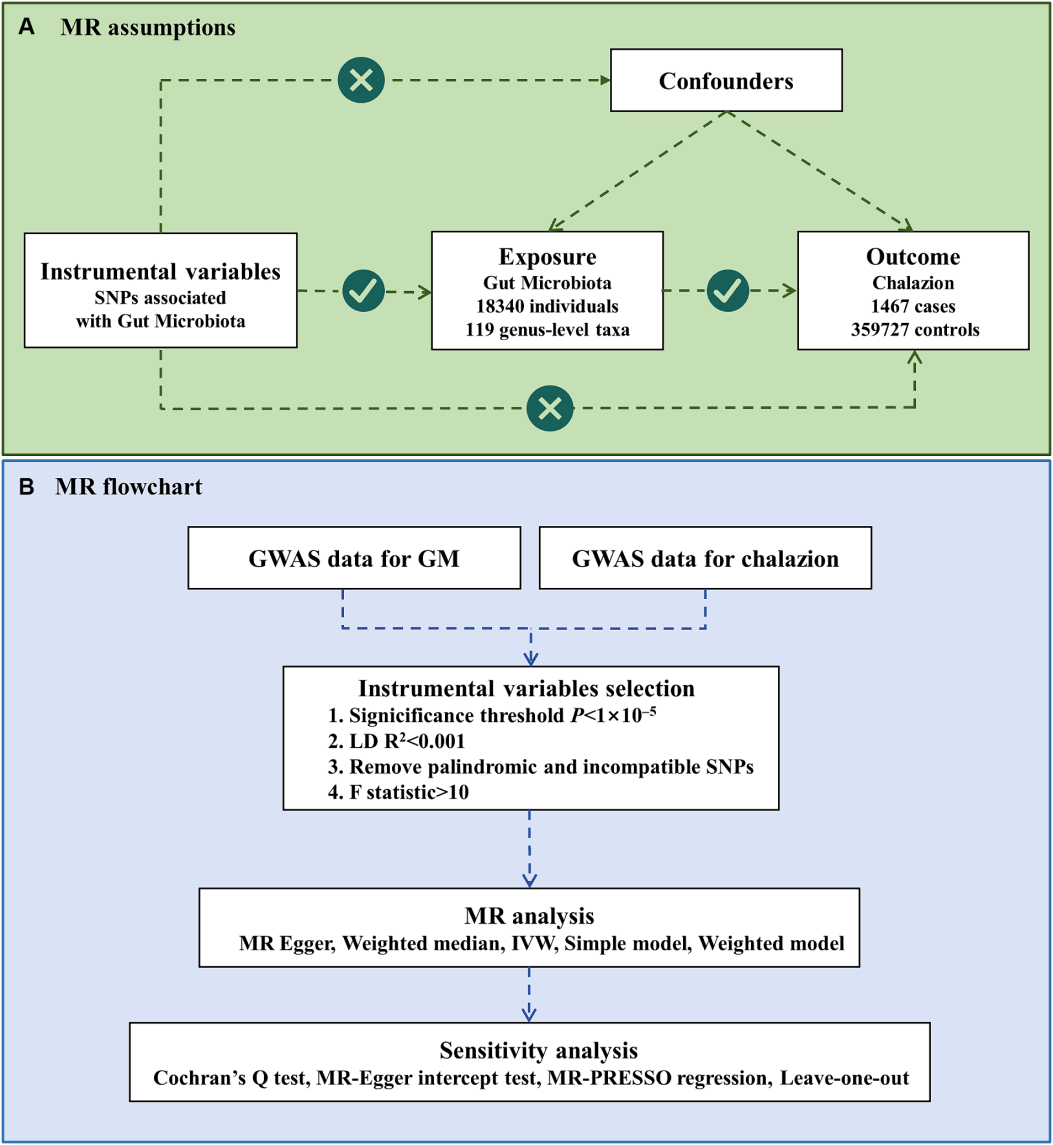
MR assumptions and flowchart of this study.

In this study, a two-sample MR analysis was performed utilizing publicly accessible summarized data derived from GWAS on GM and chalazion. The following three assumptions were satisfied to ensure the credibility of the result: (1) There existed a significant correlation between genetic variations and exposure. (2) Genetic variants chosen as instrumental variables (IVs) should be independent of confounding factors linked to both exposure and outcome. (3) There was no horizontal pleiotropy between genetic variations and outcome. By adhering to these assumptions, we effectively reduced the potential confounding effects of systemic diseases commonly associated with chalazion. Figure 1 showed MR assumptions and flowchart of this study.

### Data sources

We acquired GWAS data pertaining to GM through the International Consortium MiBioGen. The dataset encompassed 16S rRNA gene profiles from a cohort of 18,340 individuals predominantly of European ancestry, drawn from 24 cohorts spanning 11 countries. The known 119 genus-level taxa identified by microbiota quantitative trait loci (mbQTL) mapping analysis were included (15). Genetic variation for the outcome was obtained from the MRC Integrative Epidemiology Unit (IEU) Open GWAS database,^1^ comprising 1,467 cases and 359,727 controls. The initial study has obtained approval from the relevant ethics and committee. Thus, this study did not necessitate further ethical approval.

### Instrumental variables

To ensure the significance between the selected single nucleotide polymorphisms (SNPs) and exposure, we chose *p* < 1 × 10^−5^ as the threshold. Linkage disequilibrium (LD) analysis was conducted (*R*^2^ < 0.001, clumping distance = 10,000 kb) to meet the assumptions of MR and palindromic and incompatible SNPs were removed. After the harmonization process, the F statistic was calculated as follows: F = *R*^2^ × (*N*−2)/(1−*R*^2^), and *N* represented the sample size. We omitted SNPs with an *F* value below 10 (16). Subsequently, using the LDtrait Tool,^2^ we discovered that the SNPs we obtained do not show any correlation with other risk factors for outcome variable.

### Statistical analysis

This study utilized five frequently employed methodologies for MR analysis, including MR-Egger, weighted median, inverse variance weighted (IVW), simple model, and weighted model. IVW method was chosen as the primary approach for estimating causal effects, utilizing Wald ratio estimates for all relevant IVs without considering the presence of intercept terms in regression. Other methods were employed as supplementary tests to ensure the reliability and stability of the results (17).

Cochran’s Q test was applied to detect heterogeneity, while the MR-Egger intercept test and MR-PRESSO regression method were employed to evaluate horizontal pleiotropy in IVs. A *p*-value exceeding 0.05 suggested no presence of heterogeneity or pleiotropy. Leave-one-out analysis was conducted by sequentially removing SNPs to identify potentially influential ones. The analysis process was completed using R version 4.2.2.

## Result

### MR analysis

**Figure 2:**
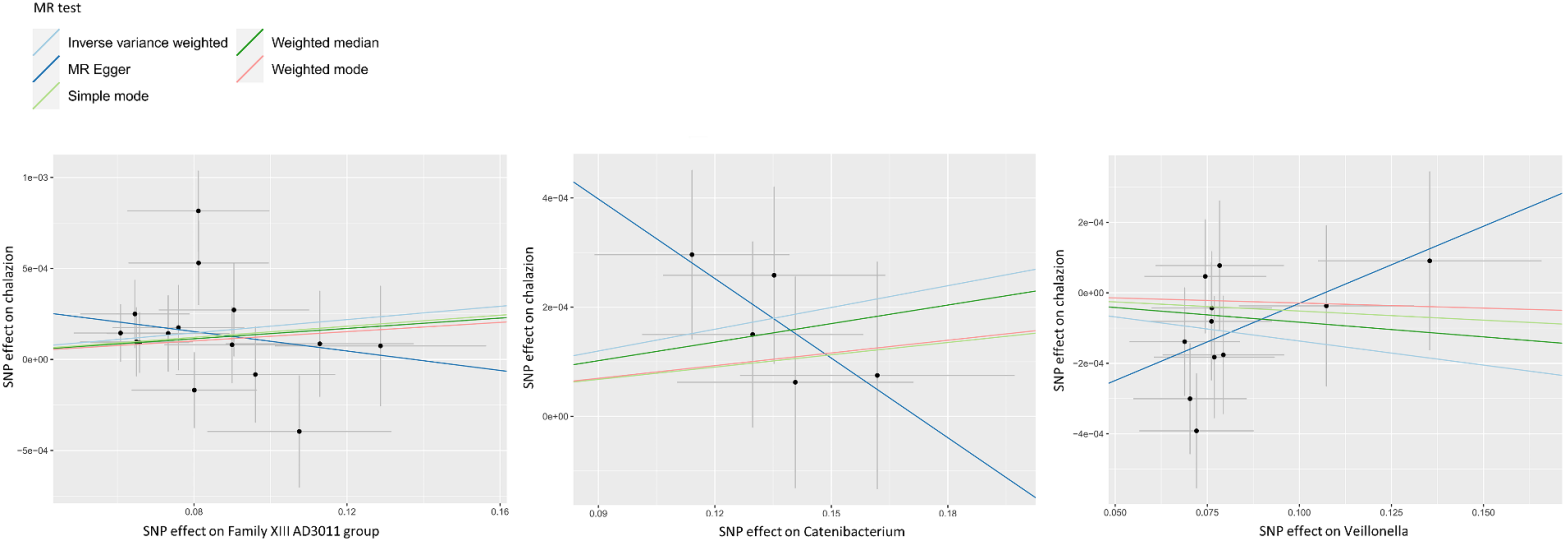
Scatter plots for the casual effect between GM and chalazion. Scatter plots for the casual effect between Family XIII AD3011 group, Catenibacterium, Veillonella and chalazion. Black points corresponded to SNPs, with their effects on exposure plotted on the horizontal axis and effects on outcome on the vertical axis. The gray lines passing through the black points represented the 95% CI.

Supplementary Table S1 provided detailed information on 1,509 SNPs in 119 genera selected as IVs (*p* < 1 × 10^−5^), with all *F*-values exceeding 10. IVW estimate indicated that Family XIII AD3011 group (OR = 1.0018, 95% CI 1.0002–1.0035, *p* = 0.030) and Catenibacterium (OR = 1.0013, 95% CI 1.0002–1.0025, *p* = 0.022) may be risk factors for chalazion. In contrast, Veillonella (OR = 0.9986, 95% CI 0.9974–0.9999, *p* = 0.036) conferred protection against chalazion (Table 1). Figure 2 showed the scatter plots for the casual effect between GM and chalazion. Supplementary Table S2 presented the MR estimates of other bacteria on the outcome.

**Table 1:** MR estimate for the association between GM and outcome.

Bacterial genera (exposures)	Methods	*N* (SNP)	*p*-value	OR	95% CI
Family XIIIAD 3011 group	MR Egger	15	0.522	0.9973	0.9894–1.0053
	Weighted median	15	0.134	1.0014	0.9996–1.0033
	IVW	15	0.030	1.0018	1.0002–1.0035
	Simple mode	15	0.353	1.0015	0.9984–1.0046
	Weighted mode	15	0.408	1.0013	0.9983–1.0042
Catenibacterium	MR Egger	5	0.424	0.9952	0.9849–1.0055
	Weighted median	5	0.142	1.0011	0.9996–1.0026
	IVW	5	0.022	1.0013	1.0002–1.0025
	Simple mode	5	0.524	1.0008	0.9986–1.0029
	Weighted mode	5	0.512	1.0008	0.9987–1.0029
Veillonella	MR Egger	11	0.260	1.0044	0.9973–1.0116
	Weighted median	11	0.328	0.9992	0.9975–1.0008
	IVW	11	0.036	0.9986	0.9974–0.9999
	Simple mode	11	0.715	0.9995	0.9968–1.0022
	Weighted mode	11	0.839	0.9997	0.9970–1.0024

CI, confidence interval; GM, gut microbiota; IVW, inverse variance weighted; MR, Mendelian randomization; SNP, single nucleotide polymorphisms.

### Sensitivity analysis

**Figure 3:**
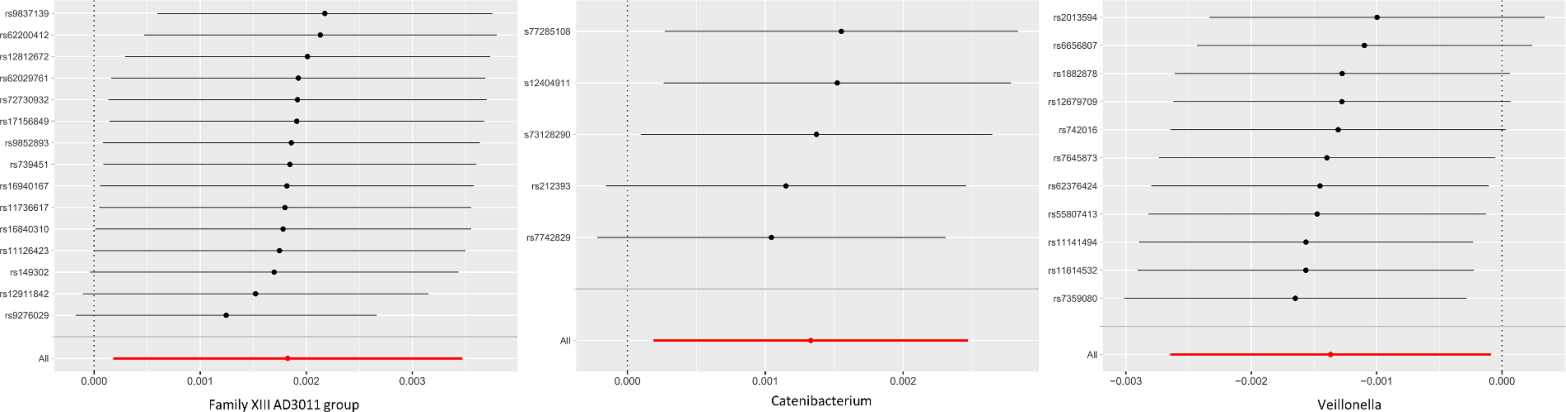
MR leave-one-out analysis for GM and chalazion. Leave-one-out analysis for Family XIII AD3011 group, Catenibacterium, Veillonella and chalazion. This method systematically excluded each SNP and conducting MR analysis to assess if the causal effects were influenced by a single SNP.

There was no heterogeneity in Family XIII AD3011 group (IVW *p* = 0.126, MR-Egger *p* = 0.145), Catenibacterium (IVW *p* = 0.738, MR-Egger *p* = 0.900), and Veillonella (IVW *p* = 0.552, MR-Egger *p* = 0.718) for chalazion. No horizontal pleiotropy was detected in these genera. MR-PRESSO *P* was 0.162, 0.766, and 0.575 for Family XIII AD3011 group, Catenibacterium, and Veillonella, respectively (Table 2). As shown in Figure 3, the leave-one-out method was employed to validate the above conclusion by removing each SNP and repeating the MR analysis on the other SNPs. Supplementary Table S3 showcased the outcomes of the analysis examining heterogeneity and pleiotropy across all bacterial genera.

**Table 2:** Sensitivity analysis between GM and chalazion.

Exposures	IVW_Q	IVW_Q_*P*	MR_Egger_Q	MR_Egger_Q_*P*	Egger_intercept	Pleiotropy_*P*	MR_PRESSO_*P*
Family XIIIAD 3011 group	20.135	0.126	18.329	0.145	0.0004	0.278	0.162
Catenibacterium	1.987	0.738	0.586	0.900	0.0008	0.322	0.766
Veillonella	8.794	0.552	6.217	0.718	−0.0005	0.143	0.575

## Discussion

Chalazion is a prevalent eyelid disorder impacting individuals across various age groups, and still lacks comprehensive understanding of its pathological mechanisms. Although investigations into the ocular surface and eyelid microbiota have been conducted, research specifically on the microbiota associated with chalazion remains scarce (18). It was speculated that the pathogenesis of chalazion may be similar to acne vulgaris or chronic blepharitis, where alterations in bacterial components modified the lipid composition of secretions, further triggering inflammation and obstruction of the sebaceous glands (2). Therefore, it was imperative to systematically investigate the microbiome dynamics of chalazion. In this study, we primarily focused on the causal role of the GM on chalazion. Via MR analysis, we probed the causal nexus between GM and chalazion, elucidating insights from a host genetics viewpoint, and corroborated the impact of GM on chalazion.

We used a threshold of *p* < 1 × 10^−5^ to obtain sufficient IVs to ensure the reliability of the results. Moreover, the value was determined as the optimal threshold for selecting genetic predictors linked to GM, as it resulted in a higher explained variance and had been utilized across numerous MR studies focused on GM (19, 20). This study employed five methods for MR analysis and IVW method served as the primary analysis. The remaining four methods were supplementary. The results of the MR-Egger method differed from those of other methods. We believed this discrepancy might be attributed to differences in calculation and the influence of publication bias. The MR-Egger considered the presence of the intercept term in regression. If the intercept term of MR-Egger differed significantly from zero, it suggested potential horizontal pleiotropy in the IVs. Therefore, we employed MR-Egger intercept test and MR-PRESSO regression to exclude the presence of horizontal pleiotropy, ensuring the robustness of the outcomes. Additionally, the MR-Egger method was sensitive to publication bias, meaning studies with significant results were more likely to be published. If there was publication bias among the included studies, it might affect the results of the MR-Egger method, leading to inconsistencies with other methods (21). The IVW results revealed that Family XIII AD3011 group and Catenibacterium genera was linked to a heightened susceptibility to chalazion, whereas Veillonella was associated with a diminished risk of chalazion. The OR identified in our study, whether indicating increased or reduced risk, were relatively small. This was not uncommon in studies involving complex traits such as GM composition and chalazion, where multiple genetic and environmental factors played a role. The large sample sizes used in this study enhanced the statistical power, allowing for the detection of even minor associations. While the OR might seem modest, their statistical significance suggested that these associations were not due to random variation. It was important to note that small effect sizes were typical in genetic epidemiology and could still be biologically meaningful. The cumulative effect of multiple small risk factors could significantly impact disease risk. Therefore, understanding the contributions of individual bacterial genera, as well as their potential interactions, was crucial for elucidating the pathogenesis of chalazion. These discoveries have the potential to inform the advancement of innovative preventive and therapeutic interventions for chalazion. Our findings underscored the need for further research to explore these relationships and their implications for therapeutic interventions.

Firmicutes and Bacteroidetes make up the predominant portion of GM, rendering their modifications especially noteworthy across various pathological states (22). Catenibacterium, belonging to the phylum Firmicutes, is a gram-positive anaerobic bacterium capable of fermenting glucose to produce short-chain fatty acids, with a higher proportion in obese individuals (23). Previous studies have shown the detrimental impacts of obesity and elevated serum cholesterol on chalazion. Our research provided evidence for this conclusion from a genetic perspective. Moreover, studies have suggested the involvement of this genus in the development of ocular diseases such as Behçet’s disease (24), allergic conjunctivitis (25), and Demodex blepharitis (26). It was hypothesized that this genus may be associated with the differentiation of Th1 cells controlled by IL-6.

Within the Firmicutes phylum, the Family XIII AD3011 group is a classification with unclear characteristics. Previous literature suggested that the gut genus Family XIII AD3011 group exhibited a heightened susceptibility to abdominal aortic aneurysm and was linked to polycystic ovary syndrome, chronic schizophrenia, and telomere shortening (27–30). There was currently no research on its involvement in ocular diseases. Additional research was required to validate the precise mechanism by which Family XIII AD3011 group contribute to chalazion.

Veillonella is a gram-negative anaerobic coccus, which along with Corynebacterium, Pseudomonas, and others, collectively constitute the core microbiota of the normal ocular surface (31). It is also a component of the normal microbiota in the oral cavity, urogenital system, respiratory tract, and intestines of both humans and animals. In recent years, the significant impact of Veillonella on the human microbiome, infection, and immune development has been elucidated. It may serve a protective and supportive function in the initial development of children’s immune systems. For instance, among children predisposed to asthma, there was a notable reduction in the abundance of Veillonella in the gut (32). However, some studies have also indicated an increase in the gut genus Veillonella in conditions such as dry eye and acute anterior uveitis (33, 34). Research on this genus in ocular conditions was limited, and our study suggested its potential protective role in chalazion.

Substantial evidence has confirmed the existence of the gut-eye axis. For instance, research has shown that CD-25 gene knockout mice exhibit symptoms resembling Sjögren’s dry eye, accompanied by alterations in microbiota composition, whereas these symptoms were mitigated after fecal transplantation (35, 36). Peng et al. demonstrated that the outer blood-retinal barrier and colonic epithelial barrier were impaired in murine with the Rd8 mutation of Crb1, facilitating the migration of GM from the lower gastrointestinal tract to the retina and secondary retinal degeneration. Systemic bacterial clearance or colonic reintroduction of normal Crb1 expression effectively mitigated retinal degeneration associated with the Rd8 mutation (37). Filippelli et al. found that supplementing microbiota could lead to complete regression of chalazion in a shorter time compared to drug therapy, suggesting the microbiome as a viable therapeutic target for ocular diseases and emphasizing the regulatory role of the gut-eye axis (12). There were currently multiple explanations. Some researchers suggested that the GM may produce substances that uphold the robustness of the intestinal barrier, regulate cytokine produced by immune cells, or act as microbial signaling molecules to generate a B cell reservoir at extraintestinal sites. Others suggested that there may be crosstalk between microRNAs present in body fluids and the microbiota to facilitate the function of the gut-eye axis (38–40).

However, no reports have explored the causal link between GM and chalazion. Our study, based on two-sample MR, indicated a correlation between the onset of chalazion and GM for the first time. This suggested a potential association between chalazion development and systemic alterations caused by abnormal GM, laying a foundation for future research. Combining our MR analysis with recent comparative pilot studies, we believed that understanding the effects of specific GM on chalazion might offer additional therapeutic avenues to enhance the efficacy of conservative treatments and potentially avoid or delay surgery. Our study was expected to provide new insights and intervention strategies for future research on chalazion.

This article also had some limitations. Although MR studies effectively minimized confounding factors through their design, complete elimination remained unattainable. Residual confounding factors might persist due to unmeasured variables or intricate interactions between genetic elements and environmental factors. To mitigate this in future investigations, we suggested utilizing more comprehensive datasets containing detailed clinical information about patients’ comorbidities. These detailed datasets could facilitate more sophisticated analyses, enabling adjustments for a wider array of confounding factors. Moreover, because chalazion were localized to the eyes and had minimal influence on GM, reverse MR analysis was not conducted owing to inadequate IVs. In addition, we acknowledged that another limitation of our study was the reliance on MR analysis, which assumed a direct correlation between host genotype and microbiota composition. While MR helped minimize confounding factors, it did not fully account for the substantial influence of environmental factors on microbiota composition. The direct correlation between host genotype and microbiota remained controversial and warranted further investigation.

## Conclusion

This MR analysis revealed the causal effects of GM on chalazion, identifying Catenibacterium and Family XIII AD3011 group as potential risk factors, while Veillonella emerged as a protective factor. Additional investigation was required to delve into the potential of manipulating GM to enhance chalazion management. Translating these findings into practical applications may allow us to exploit the benefits of regulating GM for chalazion management and recurrence reduction.

## Data availability statement

The original contributions presented in the study are included in the article/Supplementary material, further inquiries can be directed to the corresponding authors.

## Ethics statement

Ethical approval was not required for the study involving humans in accordance with the local legislation and institutional requirements. Written informed consent to participate in this study was not required from the participants or the participants’ legal guardians/next of kin in accordance with the national legislation and the institutional requirements.

## Author contributions

WZ: Writing – original draft. XG: Writing – review & editing, Methodology. QZ: Writing – review & editing, Methodology. CW: Writing – review & editing, Investigation. XL: Writing – review & editing, Investigation. YC: Writing – review & editing, Project administration. XZ: Writing – review & editing, Methodology.

## Footnotes

^1^ http://gwas.mrcieu.ac.uk

^2^ https://ldlink.nih.gov/?tab=ldtrait

## References

[1] Suzuki T Katsuki N Tsutsumi R Uchida K Ohashi K Eishi Y. Reconsidering the pathogenesis of chalazion. Ocul Surf. (2022) 24:31–3. doi: 10.1016/j.jtos.2021.12.010, PMID: 34958961

[2] Kim ES Afshin EE Elahi E. The lowly chalazion. Surv Ophthalmol. (2023) 68:784–93. doi: 10.1016/j.survophthal.2022.11.002, PMID: 36395826

[3] Evans J Vo KBH Schmitt M. Chalazion: racial risk factors for formation, recurrence, and surgical intervention. Can J Ophthalmol. (2022) 57:242–6. doi: 10.1016/j.jcjo.2021.04.023, PMID: 34062122 PMC9161235

[4] Patel S Tohme N Gorrin E Kumar N Goldhagen B Galor A. Prevalence and risk factors for chalazion in an older veteran population. Br J Ophthalmol. (2022) 106:1200–5. doi: 10.1136/bjophthalmol-2020-318420, PMID: 33789846 PMC8481354

[5] Kalyana Chakravarthy S Jayasudha R Sai Prashanthi G Ali MH Sharma S Tyagi M. Dysbiosis in the gut bacterial microbiome of patients with uveitis, an inflammatory disease of the eye. Indian J Microbiol. (2018) 58:457–69. doi: 10.1007/s12088-018-0746-9, PMID: 30262956 PMC6141402

[6] Iovieno A Lambiase A Sacchetti M Stampachiacchiere B Micera A Bonini S. Preliminary evidence of the efficacy of probiotic eye-drop treatment in patients with vernal keratoconjunctivitis. Graefes Arch Clin Exp Ophthalmol. (2008) 246:435–41. doi: 10.1007/s00417-007-0682-6, PMID: 18040708

[7] Tavakoli A Flanagan JL. The case for a more holistic approach to dry eye disease: is it time to move beyond antibiotics? Antibiotics (Basel). (2019) 8:88. doi: 10.3390/antibiotics8030088 31262073 PMC6783892

[8] Lin P. The role of the intestinal microbiome in ocular inflammatory disease. Curr Opin Ophthalmol. (2018) 29:261–6. doi: 10.1097/ICU.0000000000000465 29538183

[9] Baim AD Movahedan A Farooq AV Skondra D. The microbiome and ophthalmic disease. Exp Biol Med (Maywood). (2019) 244:419–29. doi: 10.1177/1535370218813616 30463439 PMC6546998

[10] Cavuoto KM Banerjee S Galor A. Relationship between the microbiome and ocular health. Ocul Surf. (2019) 17:384–92. doi: 10.1016/j.jtos.2019.05.006 31125783

[11] Lin P. Importance of the intestinal microbiota in ocular inflammatory diseases: a review. Clin Experiment Ophthalmol. (2019) 47:418–22. doi: 10.1111/ceo.13493, PMID: 30834680

[12] Filippelli M dell’Omo R Amoruso A Paiano I Pane M Napolitano P. Intestinal microbiome: a new target for chalaziosis treatment in children? Eur J Pediatr. (2021) 180:1293–8. doi: 10.1007/s00431-020-03880-5, PMID: 33226501

[13] Filippelli M dell’Omo R Amoruso A Paiano I Pane M Napolitano P. Effectiveness of oral probiotics supplementation in the treatment of adult small chalazion. Int J Ophthalmol. (2022) 15:40–4. doi: 10.18240/ijo.2022.01.06, PMID: 35047354 PMC8720352

[14] Sekula P Del Greco MF Pattaro C Köttgen A. Mendelian randomization as an approach to assess causality using observational data. J Am Soc Nephrol. (2016) 27:3253–65. doi: 10.1681/ASN.2016010098, PMID: 27486138 PMC5084898

[15] Kurilshikov A Medina-Gomez C Bacigalupe R Radjabzadeh D Wang J Demirkan A. Large-scale association analyses identify host factors influencing human gut microbiome composition. Nat Genet. (2021) 53:156–65. doi: 10.1038/s41588-020-00763-1, PMID: 33462485 PMC8515199

[16] Feng R Lu M Xu J Zhang F Yang M Luo P. Pulmonary embolism and 529 human blood metabolites: genetic correlation and two-sample Mendelian randomization study. BMC Genom Data. (2022) 23:69. doi: 10.1186/s12863-022-01082-6, PMID: 36038828 PMC9422150

[17] Bowden J Davey Smith G Haycock PC Burgess S. Consistent estimation in Mendelian randomization with some invalid instruments using a weighted median estimator. Genet Epidemiol. (2016) 40:304–14. doi: 10.1002/gepi.21965 27061298 PMC4849733

[18] Gomes JÁP Frizon L Demeda VF. Ocular surface microbiome in health and disease. Asia Pac J Ophthalmol (Phila). (2020) 9:505–11. doi: 10.1097/APO.0000000000000330, PMID: 33323705

[19] Brown K Godovannyi A Ma C Zhang Y Ahmadi-Vand Z Dai C. Prolonged antibiotic treatment induces a diabetogenic intestinal microbiome that accelerates diabetes in NOD mice. ISME J. (2016) 10:321–32. doi: 10.1038/ismej.2015.114, PMID: 26274050 PMC4737925

[20] Sanna S van Zuydam NR Mahajan A Kurilshikov A Vich Vila A Võsa U. Causal relationships among the gut microbiome, short-chain fatty acids and metabolic diseases. Nat Genet. (2019) 51:600–5. doi: 10.1038/s41588-019-0350-x, PMID: 30778224 PMC6441384

[21] Burgess S Thompson SG. Interpreting findings from Mendelian randomization using the MR-egger method. Eur J Epidemiol. (2017) 32:377–89. doi: 10.1007/s10654-017-0255-x, PMID: 28527048 PMC5506233

[22] Ling Z Liu X Cheng Y Yan X Wu S. Gut microbiota and aging. Crit Rev Food Sci Nutr. (2022) 62:3509–34. doi: 10.1080/10408398.2020.1867054 33377391

[23] Pinart M Dötsch A Schlicht K Laudes M Bouwman J Forslund SK. Gut microbiome composition in obese and non-obese persons: a systematic review and Meta-analysis. Nutrients. (2021) 14:12. doi: 10.3390/nu14010012, PMID: 35010887 PMC8746372

[24] Yasar Bilge NS Pérez Brocal V Kasifoglu T Bilge U Kasifoglu N Moya A. Intestinal microbiota composition of patients with Behçet’s disease: differences between eye, mucocutaneous and vascular involvement. The Rheuma-BIOTA study. Clin Exp Rheumatol. (2020) 38:60–8. 33124578

[25] Liu K Cai Y Song K Yuan R Zou J. Clarifying the effect of gut microbiota on allergic conjunctivitis risk is instrumental for predictive, preventive, and personalized medicine: a Mendelian randomization analysis. EPMA J. (2023) 14:235–48. doi: 10.1007/s13167-023-00321-9, PMID: 37275551 PMC10201039

[26] Zou D Lu X Song F Zhong X Chen H Zhang J. Characteristics of bacterial community in eyelashes of patients with Demodex blepharitis. Parasit Vectors. (2024) 17:64. doi: 10.1186/s13071-024-06122-x, PMID: 38355686 PMC10868039

[27] Zhou X Ruan W Wang T Liu H Du L Huang J. Exploring the impact of gut microbiota on abdominal aortic aneurysm risk through a bidirectional Mendelian randomization analysis. J Vasc Surg. (2024):79763–775.e2. doi: 10.1016/j.jvs.2023.11.041 38042512

[28] Lüll K Arffman RK Sola-Leyva A Molina NM Aasmets O Herzig KH. The gut microbiome in polycystic ovary syndrome and its association with metabolic traits. J Clin Endocrinol Metab. (2021) 106:858–71. doi: 10.1210/clinem/dgaa848, PMID: 33205157

[29] Chen SS Liao XM Wei QZ Zhou YY Su MY Hu Y. Associations of the gut microbiota composition and fecal short-chain fatty acids with leukocyte telomere length in children aged 6 to 9 years in Guangzhou, China: a cross-sectional study. J Nutr. (2022) 152:1549–59. doi: 10.1093/jn/nxac063, PMID: 35278080

[30] Misiak B Pawlak E Rembacz K Kotas M Żebrowska-Różańska P Kujawa D. Associations of gut microbiota alterations with clinical, metabolic, and immune-inflammatory characteristics of chronic schizophrenia. J Psychiatr Res. (2024) 171:152–60. doi: 10.1016/j.jpsychires.2024.01.036, PMID: 38281465

[31] Huang Y Yang B Li W. Defining the normal core microbiome of conjunctival microbial communities. Clin Microbiol Infect. (2016) 22:643.e7–643.e12. doi: 10.1016/j.cmi.2016.04.008, PMID: 27102141

[32] Arrieta MC Stiemsma LT Dimitriu PA Thorson L Russell S Yurist-Doutsch S. Early infancy microbial and metabolic alterations affect risk of childhood asthma. Sci Transl Med. (2015) 7:307ra152. doi: 10.1126/scitranslmed.aab2271 26424567

[33] Huang X Ye Z Cao Q Su G Wang Q Deng J. Gut microbiota composition and fecal metabolic phenotype in patients with acute anterior uveitis. Invest Ophthalmol Vis Sci. (2018) 59:1523–31. doi: 10.1167/iovs.17-22677, PMID: 29625474

[34] Moon J Choi SH Yoon CH Kim MK. Gut dysbiosis is prevailing in Sjögren’s syndrome and is related to dry eye severity. PLoS One. (2020) 15:e0229029. doi: 10.1371/journal.pone.0229029, PMID: 32059038 PMC7021297

[35] Zavos C Kountouras J Sakkias G Venizelos I Deretzi G Arapoglou S. Histological presence of *Helicobacter pylori* bacteria in the trabeculum and iris of patients with primary open-angle glaucoma. Ophthalmic Res. (2012) 47:150–6. doi: 10.1159/000330053, PMID: 22094712

[36] Zi C Huang Q Ren Y Yao H He T Gao Y. Meibomian gland dysfunction and primary Sjögren’s syndrome dry eye: a protocol for systematic review and meta-analysis. BMJ Open. (2021) 11:e048336. doi: 10.1136/bmjopen-2020-048336, PMID: 35044322 PMC8719179

[37] Peng S Li JJ Song W Li Y Zeng L Liang Q. CRB1-associated retinal degeneration is dependent on bacterial translocation from the gut. Cell. (2024) 187:1387–1401.e13. doi: 10.1016/j.cell.2024.01.040, PMID: 38412859

[38] Trujillo-Vargas CM Schaefer L Alam J Pflugfelder SC Britton RA de Paiva CS. The gut-eye-lacrimal gland-microbiome axis in Sjögren syndrome. Ocul Surf. (2020) 18:335–44. doi: 10.1016/j.jtos.2019.10.006, PMID: 31644955 PMC7124975

[39] Kugadas A Wright Q Geddes-McAlister J Gadjeva M. Role of microbiota in strengthening ocular mucosal barrier function through secretory IgA. Invest Ophthalmol Vis Sci. (2017) 58:4593–600. doi: 10.1167/iovs.17-22119, PMID: 28892827 PMC5595225

[40] Nayyar A Gindina S Barron A Hu Y Danias J. Do epigenetic changes caused by commensal microbiota contribute to development of ocular disease? A review of evidence. Hum Genomics. (2020) 14:11. doi: 10.1186/s40246-020-00257-5, PMID: 32169120 PMC7071564

